# Transformer enhanced autoencoder rendering cleaning of noisy optical coherence tomography images

**DOI:** 10.1117/1.JMI.11.3.034008

**Published:** 2024-04-30

**Authors:** Hanya Ahmed, Qianni Zhang, Robert Donnan, Akram Alomainy

**Affiliations:** a Queen Mary University of London, School of Electronic Engineering and Computer Science, London, United Kingdom; b Queen Mary University of London, School of Engineering and Materials Science, London, United Kingdom

**Keywords:** transformers, deep learning, image denoising, optical coherence tomography, ophthalmology, dentistry

## Abstract

**Purpose:**

Optical coherence tomography (OCT) is an emerging imaging tool in healthcare with common applications in ophthalmology for detection of retinal diseases, as well as other medical domains. The noise in OCT images presents a great challenge as it hinders the clinician’s ability to diagnosis in extensive detail.

**Approach:**

In this work, a region-based, deep-learning, denoising framework is proposed for adaptive cleaning of noisy OCT-acquired images. The core of the framework is a hybrid deep-learning model named transformer enhanced autoencoder rendering (TEAR). Attention gates are utilized to ensure focus on denoising the foreground and to remove the background. TEAR is designed to remove the different types of noise artifacts commonly present in OCT images and to enhance the visual quality.

**Results:**

Extensive quantitative evaluations are performed to evaluate the performance of TEAR and compare it against both deep-learning and traditional state-of-the-art denoising algorithms. The proposed method improved the peak signal-to-noise ratio to 27.9 dB, CNR to 6.3 dB, SSIM to 0.9, and equivalent number of looks to 120.8 dB for a dental dataset. For a retinal dataset, the performance metrics in the same sequence are: 24.6, 14.2, 0.64, and 1038.7 dB, respectively.

**Conclusions:**

The results show that the approach verifiably removes speckle noise and achieves superior quality over several well-known denoisers.

## 1 Introduction

Optical coherence tomography (OCT) is a medical imaging technique that uses low-coherence infrared light to harmlessly probe into the human body.^1^ Low coherence, however, leads to speckle noise in imaging; thus, it gives rise to a poor signal-to-noise ratio (SNR), confounding the imaging detail and introducing artifacts.^2^ OCT has been widely implemented in clinical practice for ophthalmology to detect multiple retinal diseases such as diabetic retinopathy (DR)^2^ and age-related macular degeneration (AMD).^3^^,^^4^ Within preliminary research in dentistry, OCT has been examined for early carious lesions, but there are no procedures yet for adequately detecting tooth decay. Speckle noise presents a great challenge as it hinders the clinician’s ability to diagnosis in extensive detail.

To address this problem, we propose a new computational denoising framework with the implementation of a new deep learning method, namely transformer enhanced autoencoder rendering (TEAR). Its layout combines transformers and autoencoders to decrease the loss of useful data and remove the different types of noise artifacts in OCT images. This method also incorporates attention gates (AGs) to put the image through a “hard-thresholding” process to suppress the background, followed by the application of a convolutional neural network (CNN) to allow for the absences of ground-truth data. The proposed method is examined with regard to different learning rates, batch sizes, and optimizers. Also, a systematic comparison is conducted with existing state-of-the-art denoisers to demonstrate the advantages of the proposed methods for clinical practice.

In summary, the contributions of this paper are as follows.

• A novel deep learning based denoising framework that includes the TEAR method is developed.
• In TEAR, transformers are integrated into an autoencoder. This is to remove noise artifacts in the regions of interest (ROI) provided by the AG. The proposed TEAR method effectively removes noise artifacts including degraded pixels without damaging the visual quality of the images.
• A new loss function is proposed along with TEAR; it combines a sliding box, contrast-to-noise ratio (CNR), peak SNR (PSNR), and mean squared error (MSE). It compares the CNR and MSE between predicted and denoised paired images to evaluate the focused regions that the AGs choose.

## 2 Related Work

### 2.1 Denoising Methods

In previous decades, numerous image denoising approaches have been created; these range from transfer domains (e.g., dual tree complex, curvelets, etc.)^5^^,^^6^ filtering methods (e.g., non-local mean (NLM), Wiener, etc.)^7^^,^^8^ and more recently machine learning (ML) methods.^9^^,^^10^^,^^11^^,^^12^ One of the well-known denoising approaches that provides effective results is block-matching and 3D filtering (BM3D).^13^ Another distinguished method is multiscale sparsity-based tomographic denoising (MSBTD), which is further refined through segmenting the image before nonlocal denoising.^14^ Nevertheless, the main drawbacks of the traditional programming methods revolve around losing meaningful detail through extra smooth appearance or limited noise removal. Also, most of the techniques are computationally intensive.

ML, specifically deep learning (DL), methods have proven to be powerful techniques for various medical image processing tasks, such as feature extraction,^15^ classification^16^ and segmentation.^17^ Most denoising techniques revolve around different layouts of CNNs. A widely implemented DL model for segmentation is generative adversarial networks (GANs). These contain two networks: the generator and the discriminator. Each is concurrently trained.^18^ GANs have been recently implemented to denoise OCT images by integrating different denoising filters (NLM) and other DL models (Siamese, Noise2Noise) into the basic GAN architecture.^19^^,^^20^^,^^21^ Another distinguished layout was created by Zhang et al. to specifically focus on natural image denoising by integrating residual learning and batch normalization to produce a denoising CNN (DnCNN).^22^ Several fields have applied DnCNN for image restoration and denoising due to its compelling results.^23^^,^^24^ However, the applications require a large number of clean data, which is not easily accessible in certain medical fields. With OCT images especially, there are limited datasets that include clean denoised images. Autoencoders (AE) were introduced to overcome this lack of large, clean OCT images datasets to tackle unsupervised learning. The concept is achieved by the DL model learning the fundamental features of data that are essential to reconstructing the data. Therefore, AEs rely heavily on dimensionality reduction because the AE is split into the encoder and decoder. AEs have been implemented to denoise OCT images with their ground truth as the averaged image.^25^ Also, a different implementation of the AE is a shared encoder (SE).^26^ However, outputting averaged images is less effective and has a longer acquisition time.

### 2.2 Transformers

First, Bahdanau et al. in 2017 suggested an improvement from long-short term memory (LSTM) called AGs.^27^ It was mainly applied for natural language processing (NLP) and later utilized for computer vision.^28^ For CNNs, AGs were created to ensure that the model focused on a certain region to allow for optimal feature extraction for the classification goal. During testing, AGs propose and highlight important ROIs and suppress irrelevant background feature activations. The main medical image processing task that AGs have been applied to is the segmentation of breast ultrasound (BUS).^28^ Focusing on OCT images, segmentation was used for certain diseases, such as DR and AMD.^29^ All mentioned studies have only applied Tversky loss for segmenting images. Yet, it has not been successfully applied for denoising images because their main objectives revolve around segmentation and classification.

Furthermore, in 2021, Dosovitskiy et al. refined AGs and developed attention-based “transformers” that learn feature representations at a highly effective rate from encoding long-range dependencies.^30^ Transformers utilize a “multi-head” attention model to correlate short- and long-distance words in both the backward and forward directions. Therefore, it outputs positional encoding for any input within the sentences.^30^ Multiple research studies created vision transformers (ViTs) for the replacement of CNNs.^31^ Within the medical field, ViTs have been widely implemented for MRI, CT, and X-rays for image segmentation, classification, and reconstruction.^32^^,^^33^^,^^31^ There is yet to be a study that deploys ViTs for image denoising on OCT images with effective and relatively accurate results.

## 3 Methodology

**Fig. 1:**
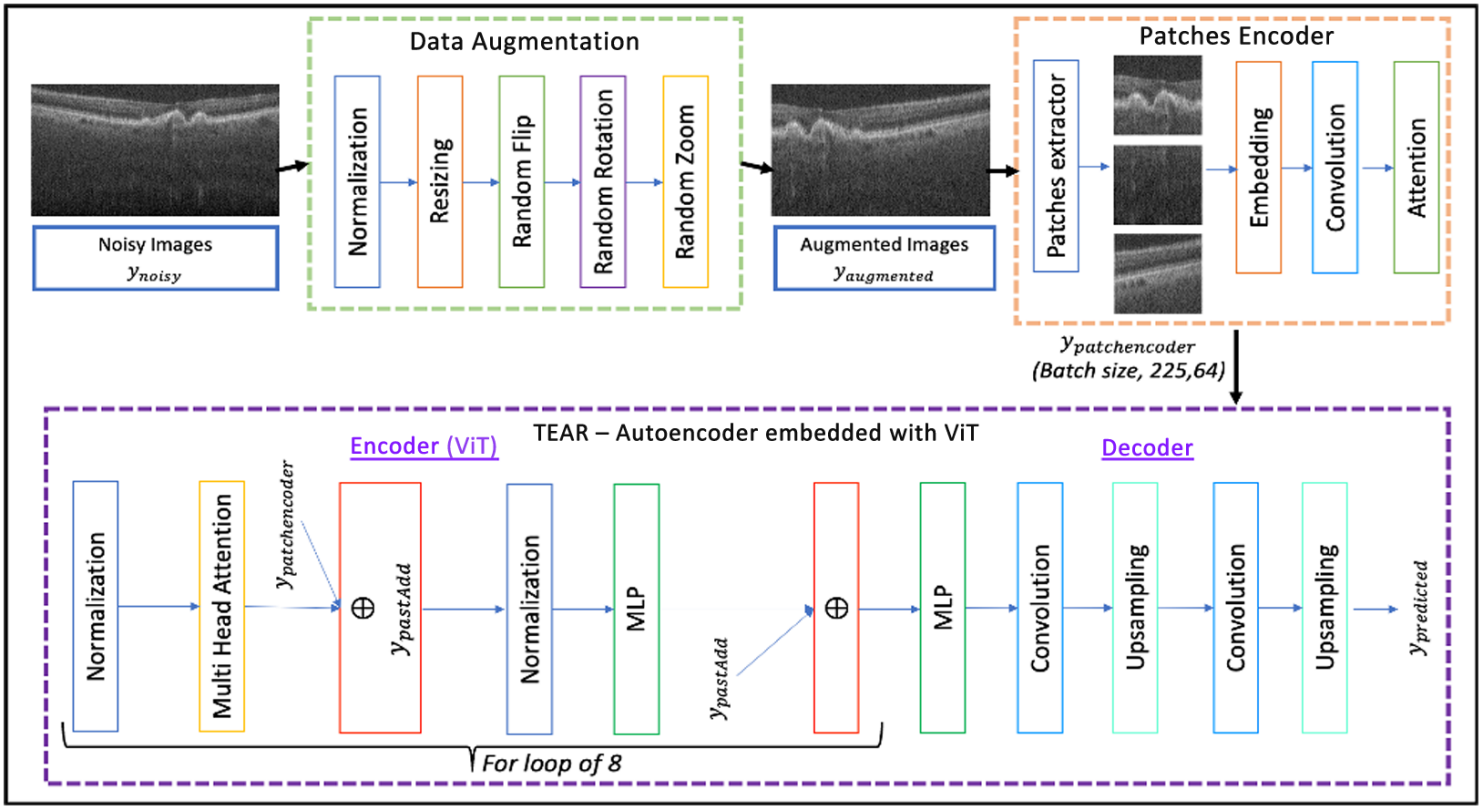
Architecture of the proposed denoising procedure. The raw images are first augmented to create a larger dataset. The augmented images are then fed into the TEAR structure, which contains an autoencoder with ViT that is managed by the loss-function during training.

In this paper, the proposed framework for OCT image denoising is described in Fig. 1. It commences with data preparation and augmentation to create more clean/noisy pairs of images. This is to overcome the main disadvantage in which there are limited clean OCT datasets for training and validation of a model. The image pairs are then augmented further to produce more pairs that are submitted as input to the CNN, which operates to remove different types of noise artifacts.

### 3.1 Data Preparation and Augmentation

To allow for light-weight CNN training, all input OCT images are first resized to 250  pixels×250  pixels. The resized images are then rotated, flipped, and enlarged to create more noisy/clean pairs to aid with the training of the framework. In addition to augmentation, patches with a size of 32  pixels×32  pixels are created from augmented images and passed through an AG. The AG converts each image-patch into fixed-length vectors of s defined size. Previous patch sizes are taken into consideration, allowing for emphasis on focused areas for the calculation of “attention scores.”

### 3.2 Attention Gate in Patch Encoder

The AG model creates scores from the input depending on ROIs that have the foreground through the application of a sliding box. It is expected to aid the network in focusing on the foreground and ignoring the background information based on the content of each image. The architecture is displayed in Fig. 1 within the patch encoder section. This consists of a few layers, starting by taking the raw image as an input ($I_{\text{input}}$) and the outputs of a sliding box with patch-sizes that represented as a query value ($I_{\text{query}}$) and are computed using Eqs. (1) and (2). Here Q is a patch matrix, and K and V are key-value image pairs. The next layers are embedding layers (E) that process both inputs and are processed to convert each image patch size into fixed-length vectors of a defined size. These layers utilize 64×64 units followed by convolution layers (*) of size 3×3 with filter sizes of 32 and 64 and a stride of 3 [Eq. (3)]. Previous patch sizes are taken into consideration, allowing for emphasis on focused areas through the query and input images to calculate matching scores. The scores create weight vectors (WS), which are processed through convolution layers to calculate the matrices of both the query and input patch sizes. The convolution outputs are processed into the attention layer that considers both the query and input patch sizes. The number of convolution units is considered to be a hyperparameter in all experiments, and it is tuned accordingly. All of the layers mentioned above are trainable and subject to change and be adjusted for different types of OCT images

$$I_{\text{input}}(Q,K,V)=\overset{N}{\underset{n=1}{\sum }}\text{softmax}(QK)\times V,$$ (1)

$$I_{\text{query}}(Q,K,V)=\overset{N}{\underset{n=1}{\sum }}\text{softmax}(QK)\times V,$$ (2)

$$\mathrm{WS}=E(A_{\text{input}})*E(A_{\text{query}}).$$ (3)

### 3.3 Transformer Enhanced Autoencoder Rendering

OCT introduces speckle noise to images; therefore, the main aim of the proposed method focuses on denoising and maintaining a rendering of the retinal and dental OCT data. As mentioned, ViT is a popular method but is mostly considered to be a medical imaging tool for segmentation and classification tasks. It has not been adapted and implemented for denoising medical images. Hence, the proposed method consists of modifying ViT and placing it as an encoder in the autoencoder. In this framework, we closely follow the original ViT,^30^ which reshapes the output of patch encoder $y_{\text{patchencoder}}$ and flattens the patches to 2D images $y_{\text{patchencoderp}}\in RN\times (P2\cdot C)$, where (P,P) is the resolution of each image patch, N is the resulting number of patches, D is the latent vector size of all layers, E is the patch embedding outputs, and C is the number of channels [Eqs. (1) and (2)]. The input is then inserted into multiple transformer blocks, containing a normalization layer at the start for computing the mean and variance along all axes of encoded patches $\left(z_{L}^{\prime }\right)$, in which $y_{\text{predicted}}$ is reshaped into the original image size

$$y_{\text{patchencoderp}}=(PE(DA(y_{\text{input}}))),$$ (4)

$$z_{0}=[y_{\text{bar}};y_{p}^{1}E;y_{p}^{2}E;\cdot ;y_{p}^{N}E]+E_{\mathrm{pos}},$$ (5)

$$E\in R^{(P^{2}\cdot C)\times D},\;E_{\mathrm{pos}}\in R^{(N+1)D},$$ (6)

$$z_{l}^{\prime }=\mathrm{MSA}(\mathrm{LN}(z_{l-1}))+z_{l-1},\;l=1,\ldots ,L,$$ (7)

$$z_{l}=\mathrm{MLP}(\mathrm{LN}(z_{l}^{\prime }))+z_{l}^{\prime },\;l=1,\ldots ,L,$$ (8)

$$y_{\text{predicted}}=\mathrm{RS}(\mathrm{LN}(z_{l}^{\prime })).$$ (9)

In the encoding process, the dimensionality of the encoded patches is adjusted to match the query dimension of the ’multi-head’ attention layer (MSA) [refer to Eq. (3)]. This meticulous adjustment ensures consistency and compatibility with the subsequent layers of the model. To maintain the independence of computations for each flattened input feature, as outlined in Eq. (5), the output of the normalization layer (LN) serves as the input for the ’multi-head’ attention layer. This layer computes attention weights based on the similarity between pairs of patches, and this process is visualized in Fig. 1. Importantly, this computation is performed across multiple heads in parallel, with the specific number of parallel heads set to 8. Each head simultaneously processes the input data, capturing diverse aspects of the relationships between patches. The outputs from these parallel heads are then concatenated to form a comprehensive and fused representation, resulting in one unified projected output. This parallelized approach enhances the model’s ability to capture intricate patterns and dependencies within the input data. The projected output is passed through another normalization layer that computes mean and variance along channels, the height and width axes of images, and then a multilayer perceptron (MLP) block [Eq. (4)]. This is to ensure that input features that are computed are completely independent of other input features of other images in a batch. The MLP block acts a classification head with Gaussian error linear units (GELUs) non-linearity.^34^ The transformer block is repeated eight times, followed by another MLP block for final encoding of the image. Finally, $y_{\text{predicted}}$ is reshaped (RS) back into the original size of the image [Eq. (6)].

**Fig. 2:**
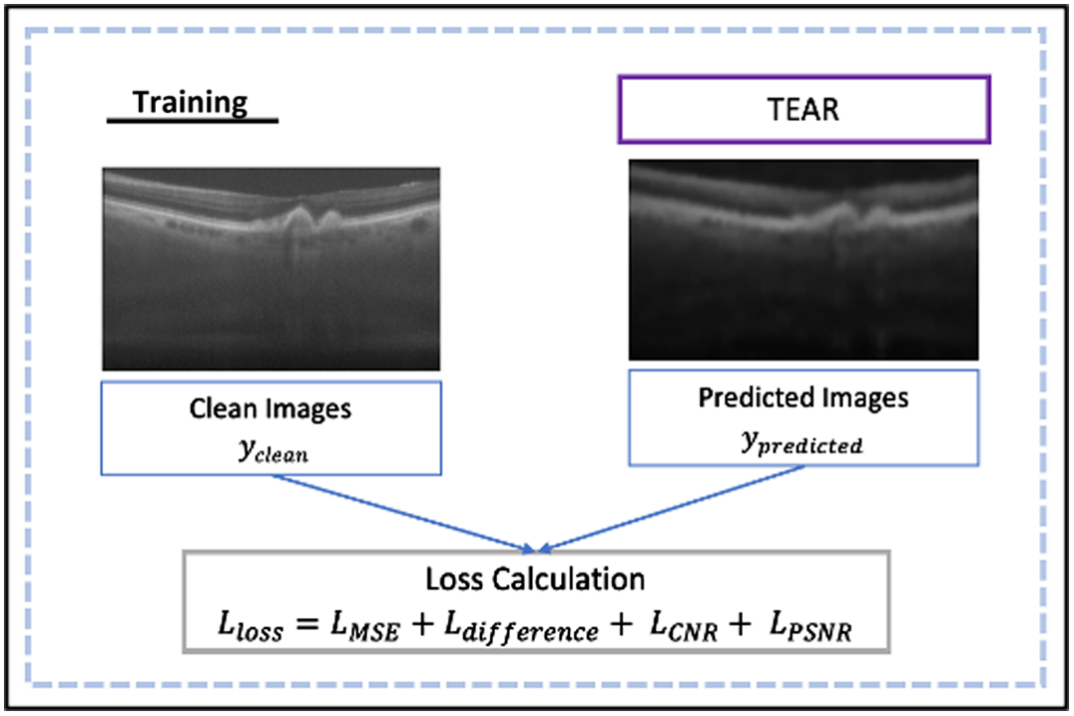
Training of TEAR with the new proposed loss function, consisting of combination of numerous image quality evaluation metrics (PSNR, CNR, and MSE) between clean and predicted images computed from TEAR.

ViT is implemented in the autoencoder as an encoder, which provides encoded weights for the decoder. This is comprised of attention scores to focus on foreground ROIs for the decoder to re-assemble the image to its full size of 500  pixels×900  pixels. The decoder consists of multiple convolution layers followed by up-sampling. The proposed model is depicted in Fig. 1. The model in the framework is evaluated with numerous learning rates $\left(5.0\times 10^{-3},1.0\times 10^{-3},1.0\times 10^{-4},1.0\times 10^{-1},1.0\times 10^{-2}\right)$; epochs (200, 500, and 1000); batch sizes (2 and 4); optimizers (ADAM, ADAMW); and image sizes (500  pixels×500  pixels, 500  pixels×900  pixels). The TEAR model is trained through the process displayed in Fig. 2 that utilizes a new proposed loss function. The environment in which the tests were implemented was Tensorflow and Keras, and all models were trained using one NVIDIA P100 GPU with 24 G memory.

### 3.4 Loss Function

**Fig. 3:**
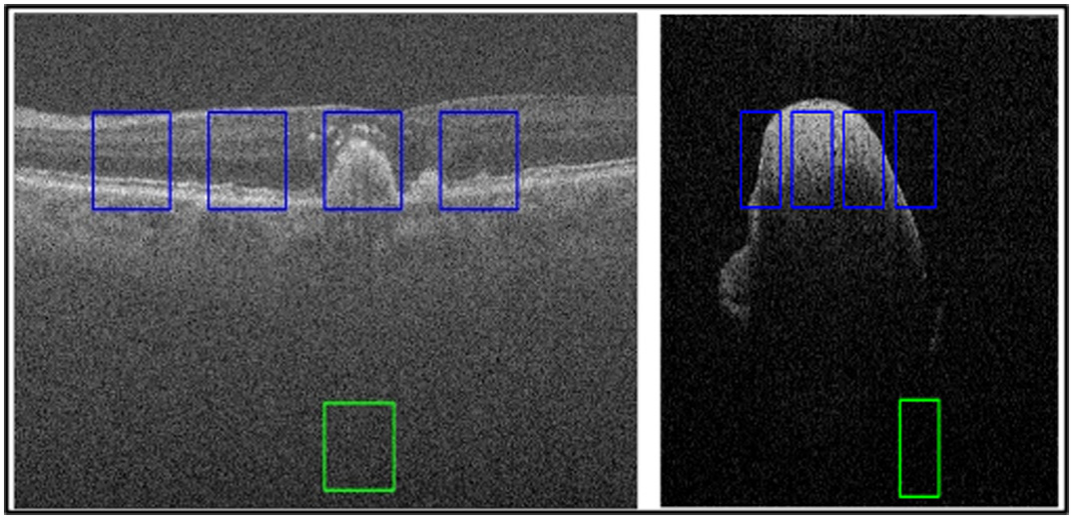
Regions of interest (ROIs) are displayed on the raw images of the DUKE (a) and dentistry (b) datasets. They are inserted into the proposed architecture for loss and evaluation calculations. Blue and green squares represent the signal and background areas, respectively.

The implementation of a combined loss function, which includes MSE, CNR, and PSNR, stems from a strategic decision to achieve a more comprehensive evaluation of denoising performance. The MSE component calculates the pixel-by-pixel differences between the predicted and ground truth images, providing an overall reconstruction accuracy measure. Including CNR in the loss function adds a perceptual quality metric, emphasizing the importance of retaining contrast information in denoised images. This inclusion ensures that the model not only reduces pixel errors but also improves the perceptual clarity of the reconstructed images. Simultaneously, the addition of PSNR enhances the evaluation by providing a standardized measure of signal fidelity. By combining these various metrics, the composite loss function allows for a more balanced optimization process, which promotes both quantitative accuracy and perceptual quality. This comprehensive approach reflects a nuanced understanding of denoising goals, encouraging the creation of models that excel not only in pixel-level fidelity but also in visual clarity and perceptual quality. The new proposed loss function is calculated through

$$L_{\text{Loss}}=L_{\mathrm{MSE}}+L_{\text{difference}}+L_{\mathrm{CNR}}+L_{\mathrm{PSNR}},$$ (10)

where $L_{\mathrm{MSE}}$ is the mean square error loss calculated while training, $L_{\text{difference}}$ is the structural similarity difference loss between predicted and actual image pairs, $L_{\mathrm{CNR}}$ is the CNR difference between actual and predicted CNR and is a normalized coefficient (0, 1), and $L_{\mathrm{PSNR}}$ is the difference of PSNR between predicted and actual image pairs that is also a normalized coefficient (0, 1). These are calculated as follows:

$$L_{\mathrm{CNR}}=1-\frac{{\mathrm{CNR}}_{A}-{\mathrm{CNR}}_{P}}{100},$$ (11)

$$L_{\mathrm{MSE}}=\frac{1}{N}\overset{N}{\underset{i=1}{\sum }}(y_{\text{actual}}-y_{\text{predicted}}{)}^{2},$$ (12)

$$L_{\mathrm{PSNR}}=1-\frac{{\mathrm{PSNR}}_{A}-{\mathrm{PSNR}}_{P}}{100}.$$ (13)

where N is the batch-size provided in training, $y_{\text{predicted}}$ is the predicted image output by the CNN, and $y_{\text{clean}}$ is the clean image. The image quality metrics are combined and normalized. The loss function aids the training of the model with a focus on ensuring that the background is set to zero for hard thresholding through the help of CNR. PSNR confirms that the image is fully reconstructed within the foreground ROIs. Therefore, during training, the model minimizes the difference between the predicted and clean image to remove noise artifacts and speckle noise that is present. Figure 3 displays a few typical OCT images from the tested datasets with the ROIs that are taken into account. For the loss function, quantitative and qualitative evaluation calculations are highlighted, with background and signal being indicated by green and blue boxes, respectively.

Subsequently, a meticulous comparative study was conducted on all datasets to rigorously evaluate the effectiveness of the proposed loss function when juxtaposed with widely employed denoising loss functions. The set of loss functions scrutinized against $L_{\text{Loss}}$ comprises traditional metrics, such as MSE, mean absolute error (MAE), and binary cross-entropy (BCE) loss. This comprehensive analysis aims to discern the nuanced performance of the proposed loss function in various denoising scenarios.

In addition to assessing loss functions, an ablation study was conducted to systematically compare the TEAR framework’s performance with and without the inclusion of additional AGs in the patch encoder. This investigation was followed by an examination of the effect of data augmentation on the framework’s overall denoising capability. These nuanced evaluations provided important insights into the role of attention mechanisms and data augmentation in improving the TEAR framework’s resilience and adaptability.

Furthermore, a thorough comparative study extended to the evaluation of the proposed framework on all datasets. This investigation sought to benchmark the TEAR framework against state-of-the-art denoising techniques, including classical methods such as BM3D,^14^ Weiner,^8^ and NLM,^7^ as well as contemporary deep learning approaches such as DnCNN,^22^ Siamese GAN,^20^ and SE.^26^ The aim was to establish the relative performance of the TEAR framework in real-world denoising scenarios, providing a comprehensive understanding of its strengths and potential advancements over existing methodologies.

### 3.5 Datasets

Two datasets from two medical fields are used to train and test the framework: a retinal OCT dataset called DUKE^14^ and a dentistry dataset collected in the QMUL IDIOT Lab (Queen Mary University of London, Institute of Dentistry in which the free space SD-OCT was set up and utilized to scan the teeth and models). Both datasets are imaged by a spectral domain OCT (SD-OCT) with an axial resolution of 4.5  μm. The DUKE consists of eighteen subjects with healthy and AMD-affected eyes; each image is 500  pixels×900  pixels. DUKE is a public dataset that provides eighteen noisy/clean pairs of images to allow for supervised learning. However, due to realignment issues, two were removed to ensure that the data is similar. The dentistry dataset consists of 10 samples with healthy and decayed teeth; each image is 500  pixels×500  pixels. Partially clean images were created for this dataset by pre-processing through basic thresholding to provide TEAR with information on foreground, so it can ignore the background. Each dataset is then randomly split into training, validation, and testing with a 60%:10%:30% split.

### 3.6 Evaluation Metrics

The proposed framework is examined using conventional image quality metrics. The equivalent number of looks (ENL) is a metric assessing the smoothing of the predicted image. It does not need a reference image because it utilizes selected ROIs of the background and signal. Figure 3 displays the ROIs utilized for calculations. ENL is defined as

$$\mathrm{ENL}=\frac{\mu_{b}^{2}}{\sigma_{s}^{2}},$$ (14)

where $\sigma_{s}$ is the standard deviation of the signal representation and $\mu_{b}$ is the mean value for background representation. PSNR provides a measure of precision of the predicted image against the clean reference image. It is calculated as

$$\mathrm{PSNR}=10\;\log(\frac{L^{2}}{\mathrm{MSE}}),$$ (15)

where L denotes the maximum possible pixel value and MSE is the mean squared error of the image. Next, structural similarity index (SSIM) is a well-known image quality metric that focuses on the perceived similarity. SSIM focuses on texture, quality degradation, and visible structures. SSIM is defined as

$$\mathrm{SSIM}=\frac{\left(2\sigma_{nc}+c_{2})(2\mu_{n}\mu_{c}+c_{1}\right)}{\left(\mu_{n}^{2}+\mu_{c}^{2}+c_{1})(\sigma_{n}^{2}+\sigma_{c}^{2}+c_{2}\right)},$$ (16)

where $\mu_{n}$, $\mu_{c}$ and $\sigma_{n}$, $\sigma_{c}$ are the mean value and standard deviation of the noisy-clean image pairs, respectively. Finally, CNR utilizes ROIs of the background and signal areas for speckle repression with respect to both areas. CNR is calculated through

$$\mathrm{CNR}=10\;\log(\frac{\mu_{s}-\mu_{b}}{\sqrt{\sigma_{b}^{2}+\sigma_{s}^{2}}}),$$ (17)

where $\mu_{s}$ and $\sigma_{s}$ are the mean value and standard deviation of the signal representation, respectively. For background representation, $\mu_{b}$ and $\sigma_{b}$ are the mean value and standard deviation.

## 4 Results

### 4.1 Ablation Study

The proposed model, TEAR, with different settings was evaluated through an evaluation study to investigate the optimal learning rates, epochs, batch size, optimizer, and image sizes. The batch size was set to four, and the optimal image size was 500  pixels×900  pixels and 500  pixels×500  pixels. The leading learning rate and epochs were $5\times 10^{-4}$ and 200, respectively, to obtain optimal results in a suitable timely manner.

**Fig. 4:**
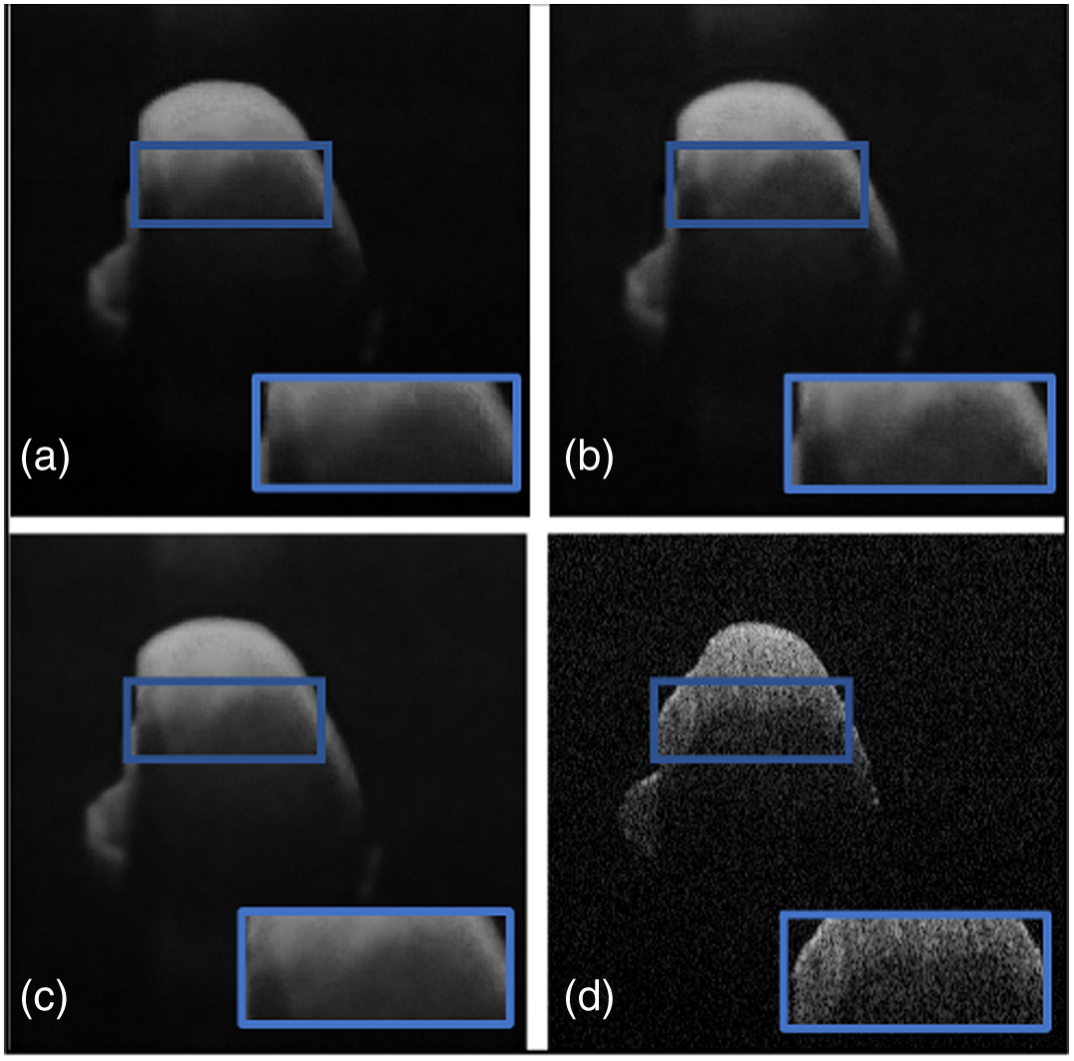
Results from ablation study for the dentistry dataset: (a) the proposed method (TEAR with $L_{\text{Loss}}$), (b) MSE loss, (c) MAE loss, and (d) BCE loss. Visual comparison is conducted, focusing on the regions in the blue boxes.

**Fig. 5:**
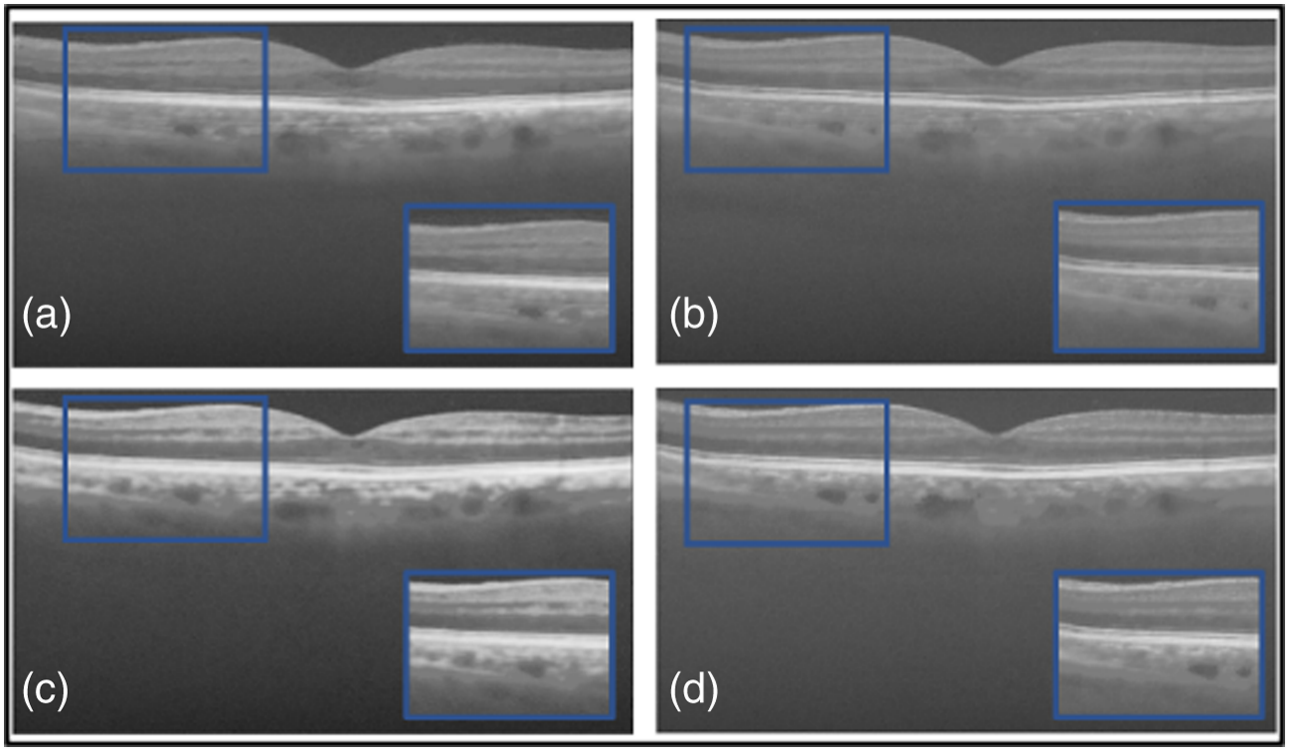
Results from ablation study for the DUKE dataset: (a) the proposed method (TEAR with $L_{\text{Loss}}$), (b) MSE loss, (c) MAE loss, and (d) BCE loss. Visual comparison is conducted, focusing on the regions in the blue boxes.

Next, a comparative study was conducted on both datasets to evaluate the proposed loss function against widely used loss functions for denoising. The loss functions examined against $L_{\text{Loss}}$ consist of MSE, MAE, and BCE loss. Table 1 shows the quantitative evaluations and leading qualitative evaluation in Figs. 4 and 5, for the dentistry and DUKE datasets, respectively. The results are averaged over the dataset. Focusing on the dentistry dataset, both the numerical and visual results showed that the MSE, MAE, and BCE losses had no positive outcome. This is displayed through SSIM results of less than 0.5 and inadequate CNR values of 3.0, 2.8, and 3.2 dB, respectively. Figure 4 shows a focused area bounded by a blue box, in which MSE [Fig. 4(a)] did not remove speckle noise thoroughly due to its presence in the blue box. However, MAE [Fig. 4(b)] produced a blurrier image than MSE indicating that speckle noise and useful data were sufficiently removed. This is shown through their PSNR results of 22.9 and 23.5 dB, respectively. BCE was not able to remove any speckle noise, confirmed by the qualitative [Fig. 4(d)] and quantitative results. The $\mathrm{TEAR}-L_{\text{Loss}}$ quantitative results display 58% and 18% higher outcomes in average SSIM and PSNR, respectively, acquiring an average of 0.64 and 24.6 dB, respectively. CNR and ENL had a twofold and fourfold increase, respectively, demonstrating a more stable speckle repression with $L_{\text{Loss}}$ than MSE, MAE, and BCE losses.

**Table 1:** Quantitative results of the proposed method with different loss functions in averaged PSNR, CNR, and ENL, all in dB, and SSIM for dentistry images.

NET	SSIM	PSNR	CNR	ENL
TEAR - $L_{\text{Loss}}$	**0.90**	**27.9**	**6.3**	**120.8**
TEAR - MSE	0.28	22.9	3.0	43.9
TEAR - MAE	0.31	23.5	2.8	43.7
TEAR - BCE	0.26	23.2	3.2	42.8

Note: bold values indicate the highest value.

With reference to the DUKE dataset, quantitative and qualitative results are displayed in Table 2 and Fig. 5. Numerically, the MAE loss obtained a CNR of 10.2 dB that is 10% higher than that of MSE and BCE, giving proof that visually it successfully thresholded the image with a darker background. However, focusing on the foreground [Fig. 5(c)] within the blue box, there is a significant addition of data between the retinal layers. This is indicated by SSIM and ENL values of 0.51 and 737.4 dB, respectively, which are a representation of low signal restoration and speckle repression compared with $\mathrm{TEAR}-L_{\text{Loss}}$ values of 0.74 and 1380.7 dB, respectively. Figures 5(b) and 5(d) display MSE and BCE outputs that have not been through sufficient thresholding, respectively. CNR values of 9.0 and 9.2 dB respectively, indicated the considerable removal of data. Shown within the blue box in Fig. 5 where the retinal data is blurred and pixelated signified by PSNR values of 22.4 and 21.8 dB, respectively. The proposed loss function ($L_{\text{Loss}}$) was able to remove any visible speckle noise as well as noise artifacts in the background. At the same time, it retained data within the retinal layers, and this is indicated by highest SSIM of 0.74 and PSNR of 24.6 dB. The difference between $L_{\text{Loss}}$ and the different loss functions in PSNR and SSIM was approximately 12% and 30%, respectively. This demonstrates the value of implementing ($L_{\text{Loss}}$) in TEAR for speckle reduction across two datasets. This is shown by the increase in all quantitative metrics for both datasets.

**Table 2:** Quantitative results of the proposed method with different loss functions in averaged PSNR, CNR, and ENL, all in dB, and SSIM for Duke images.

NET	SSIM	PSNR	CNR	ENL
TEAR - $L_{\text{Loss}}$	**0.74**	**24.6**	**14.2**	**1380.7**
TEAR - MSE	0.56	22.4	9.0	1197.7
TEAR - MAE	0.51	21.7	10.2	737.4
TEAR - BCE	0.54	21.8	9.9	1248.6

Note: bold values indicate the highest value.

**Fig. 6:**
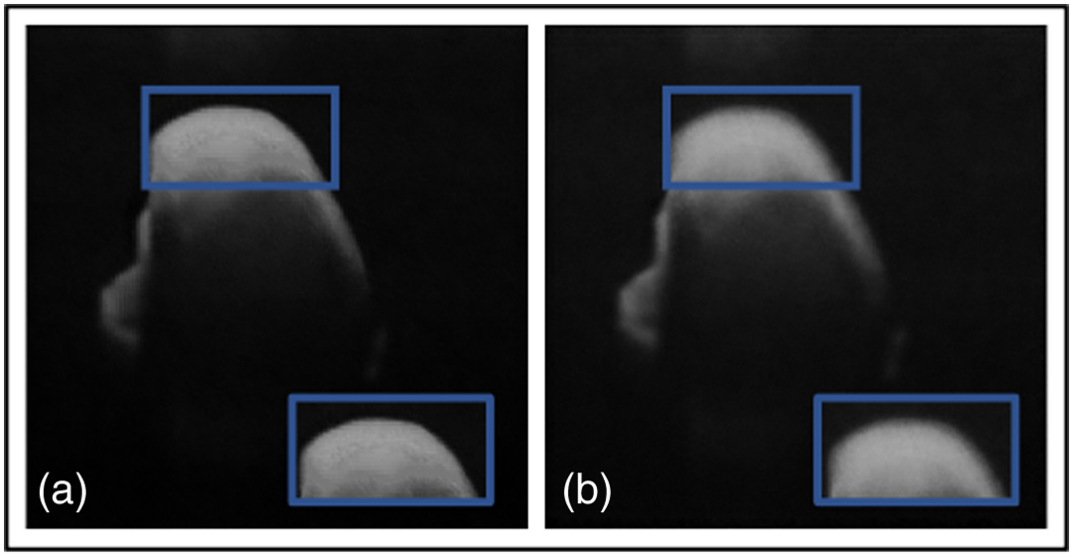
Results from ablation study for the dentistry dataset: (a) the proposed method (TEAR with $L_{\text{Loss}}$) with AGs and (b) without AGs. Visual comparison is conducted, focusing on the regions in the blue boxes.

**Fig. 7:**
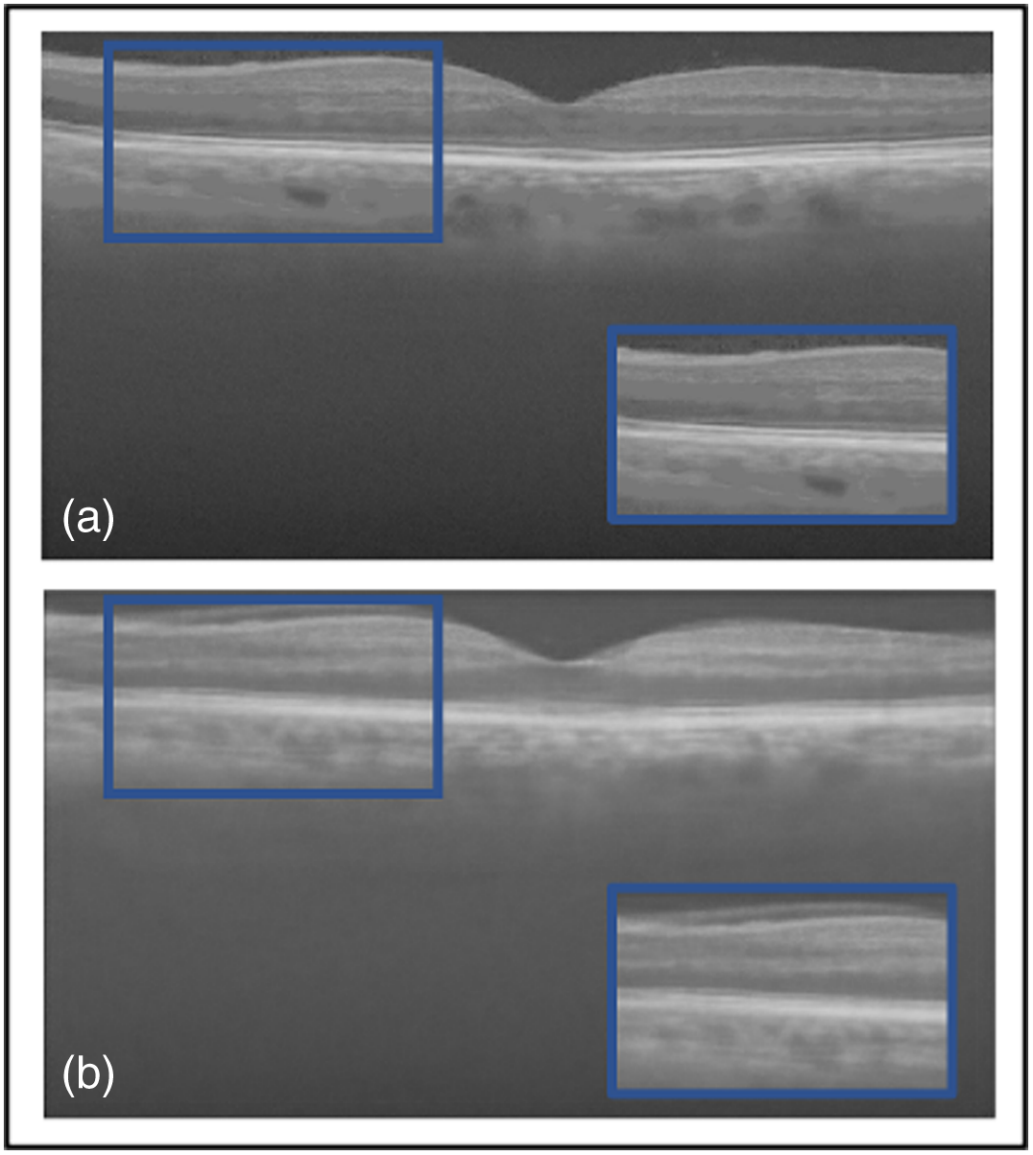
Results from ablation study for the DUKE Dataset: (a) the proposed method (TEAR with $L_{\text{Loss}}$) with AGs and (b) without AGs. Visual comparison is conducted, focusing on the regions in the blue boxes.

Another ablation study compared the TEAR framework with and without the additional AGs in the patch encoder and data augmentation. Table 3 shows the quantitative results and leading qualitative evaluation in Figs. 6 and 7 for the dentistry and DUKE datasets, respectively. Numerical results display that TEAR with AG produces a 15% and 27% increase in SSIM for the dentistry and DUKE datasets, respectively, as well as a 13% and 10% increase in PSNR for the datasets, respectively, indicating the value of AGs in signal restoration. Visually, TEAR without AGs for dentistry blurred the data at a closer look [Fig. 6(b) (blue box)] and for the DUKE dataset added layers to the retinal data [Fig. 7(b) (blue box)]. Regarding CNR and ENL for the dentistry dataset, AGs provides an average of increase of 7% and 14%, respectively. For the DUKE dataset, CNR and ENL were 26% and 22%, respectively, which demonstrates the value of implementing AGs alongside $L_{\text{Loss}}$ in TEAR for speckle reduction. This is shown by the increase in all quantitative and qualitative metrics for both datasets.

**Table 3:** Quantitative results of the proposed method with and without AGs in averaged PSNR, CNR, and ENL, all in dB, and SSIM for both datasets.

NET	SSIM	PSNR	CNR	ENL
Dentistry dataset				
TEAR - without AG	0.76	24.2	5.9	103.3
TEAR - with AG	**0.90**	**27.9**	**6.3**	**120.8**
Duke dataset				
TEAR - without AG	**0.74**	**24.6**	**14.2**	**1390.7**
TEAR - with AG	0.54	22.1	10.5	1077.0

Note: bold values indicate the highest value.

### 4.2 Comparative Study

Furthermore, a comparative study was conducted on the DUKE and dentistry datasets to examine the proposed framework against the state-of-the-art denoisers, BM3D,^14^ Weiner,^8^ and NLM,^7^ and the deep learning techniques, DnCNN,^22^ Siamese GAN,^20^ and SE.^26^ Quantitative results, with evaluation metrics for our proposed method against state-of-the-art denoisers for the DUKE dataset, are shown in Table 1. The results are averaged over the dataset.

**Fig. 8:**
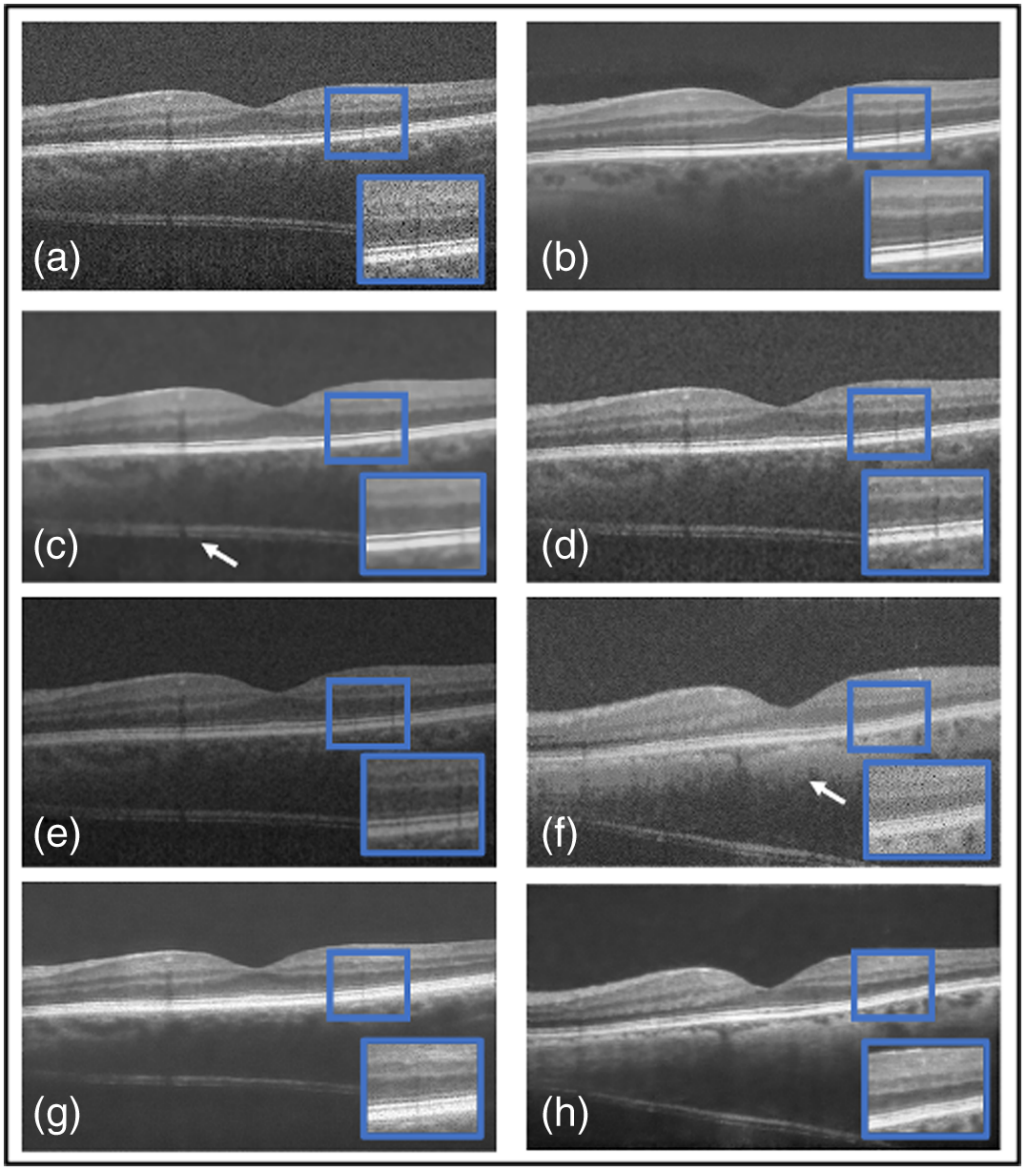
Results from the comparative study on Duke image 1: (a) the original, (b) TEAR method, (c) BM3D,^14^ (d) NLM,^7^ (e) Wiener,^8^ (f) DnCNN,^22^ (g) SiameseGAN,^20^ and (h) shared encoder (SE).^26^ Visual comparison is conducted, focusing on the regions in the blue boxes and pointed at by the white arrows.

**Fig. 9:**
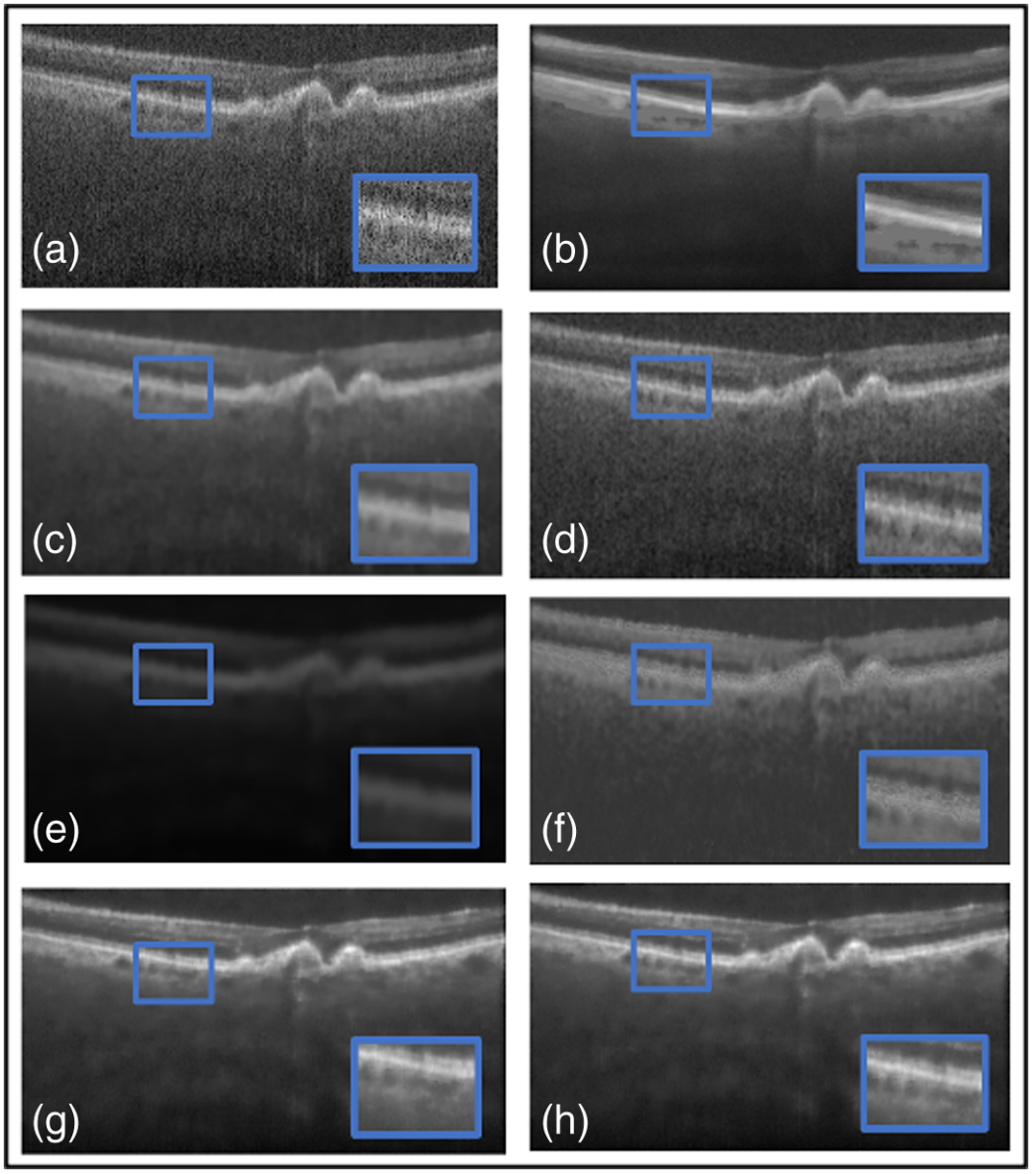
Results from the comparative study on Duke image 2: (a) the original (b) TEAR method, (c) BM3D,^14^ (d) NLM,^7^ (e) Wiener,^8^ (f) DnCNN,^22^ (g) SiameseGAN,^20^ and (h) shared encoder (SE).^26^ Visual comparison is conducted, focusing on the regions in the blue boxes and pointed at by the white arrows.

**Fig. 10:**
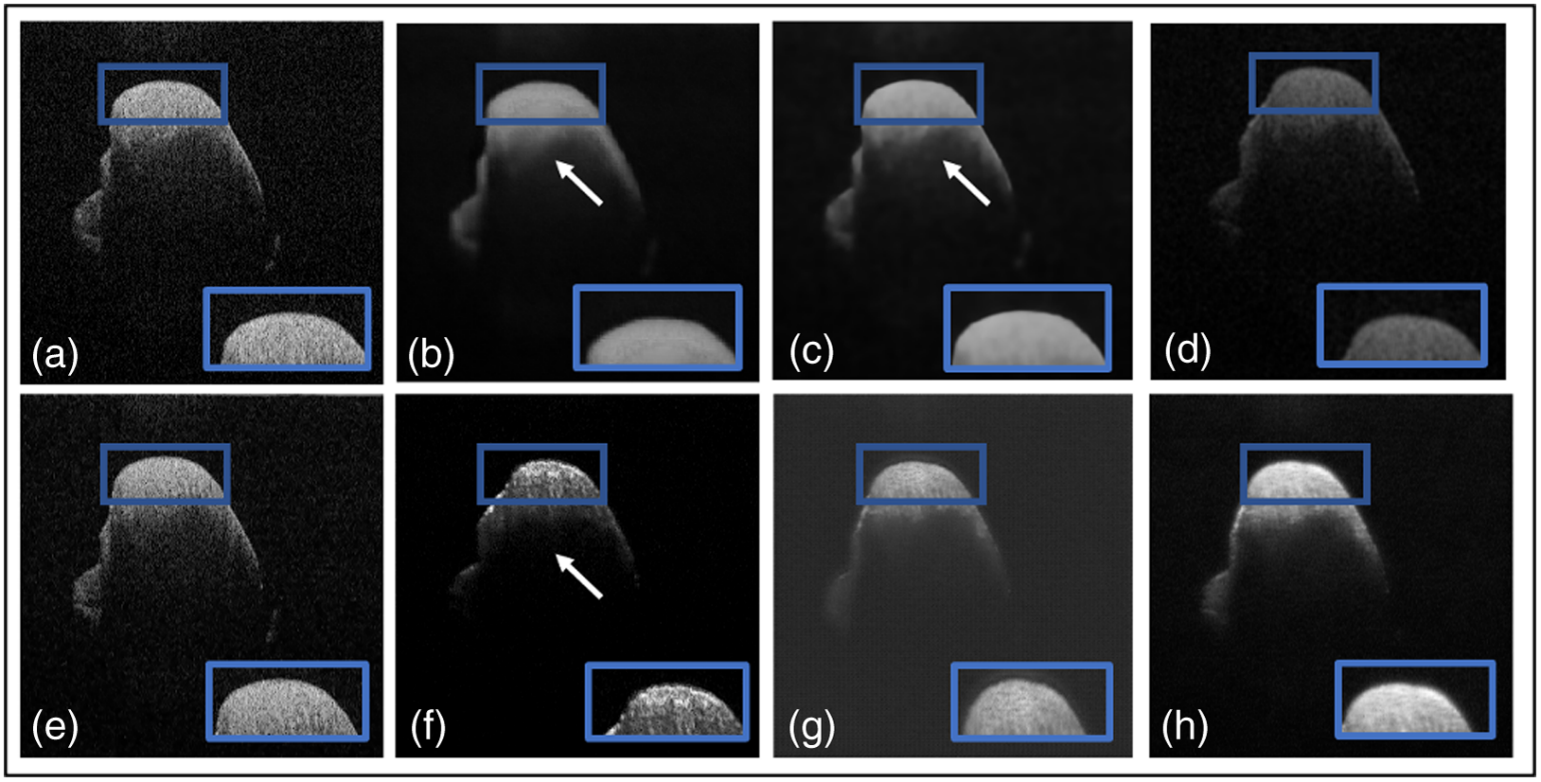
Results from the comparative study on the dentistry dataset: (a) the original, (b) TEAR method, (c) BM3D,^14^ (d) NLM,^7^ (e) Wiener,^8^ (f) DnCNN,^22^ (g) SiameseGAN,^20^ and (h) SE.^26^ Visual comparison is conducted, focusing on the regions in the blue boxes and pointed at by the white arrows.

Numerous images are shown from each dataset for a qualitative comparison of the outputs of the well-known denoisers with our proposed framework in Figs. 8–10. In the qualitative comparison with quantitative measurements, NLM and Wiener were not been able to remove any speckle noise, which is shown in Figs. 8(d) and 8(e). This is shown through their SSIM results of 0.51 and 0.53. However, Wiener was able to put the image through hard thresholding [shown in Fig 8(e)], with the darkened background. This is indicated with the relatively high CNR of 9.5 dB due to some of the foreground being taken as background. Finally, the traditional programming method of BM3D is considered. It removed the majority of noise and was able to reconstruct the gaps of data within the image [shown in the blue box in Fig. 8(c)]. The reconstruction has yet to be confirmed as useful or excess data that can be misinterpreted by clinicians. Hence it obtained the highest PSNR of 25.0 dB and second highest SSIM of 0.62. Yet it did not remove noise artifacts in the background, as indicated by the white arrows of Fig. 8(c). In the deep learning methods, DnCNN [Fig. 8(f)] reconstructed the gaps in the foreground, but speckle noise is still observable. In addition, added data, indicated by a white arrow in Fig. 8(f), have an SSIM of 0.28 dB and PSNR of 15.9 dB, confirming the qualitative conclusions. Unlike DnCNN, SiameseGAN blurred a lot of data in between the layers [shown in the blue box in Fig. 8(g)], which is indicated through the low SSIM and CNR values of 0.57 and 5.8 dB, respectively. Finally, SE provided a successful thresholded image, indicated both visually [Fig. 8(h)] and with a CNR value of 7.4 dB. However, similar to DnCNN, it reconstructed the gaps in the foreground in a pixelated manner. The proposed method was able to remove any visible speckle noise as well as noise artifacts in the background while retaining data within the retinal layers; this is indicated with the highest SSIM of 0.74 and second highest PSNR of 24.6 dB. The difference between our proposed method and BM3D in PSNR was ∼2%. Visually, SiameseGAN, BM3D, and TEAR had the leading outputs, but our proposed method had the ability to distinguish the background with its noise artifacts and remove them. In addition to BM3D, reconstruction data within the retinal layers that is possibly false data added to image. This is displayed in Fig. 8(c) within the blue box. On the other hand, SiamaseGAN blurred the area within the retinal layers, as displayed in the blue box in Fig. 8(g). However, the TEAR reconstructed the image at a limited scope to provide clinicians with an easier image to examine without removing useful data [Fig. 5(b) focused on the blue box]. The proposed method also had the leading CNR and ENL, which are performance measures of speckle repression at the specified ROIs chosen in Fig. 3.

Another image from the testing Duke dataset was evaluated qualitatively in Fig. 9. This image also contains multiple layers with speckle noise, plus a different type of noise artifacts. Two methods had the ability to remove it, our proposed method and Wiener, as shown in Figs. 9(b) and 9(e), respectively. However, Wiener once more was unable to separate the background from the foreground, thus the blurring and removal of retinal layers. Also, NLM visually did not attempt to remove any speckle noise [Fig. 9(d)]. Focusing on BM3D and the proposed method, the blue boxes in Figs. 9(b) and 9(c) display the region that explains why BM3D obtained the highest PSNR of 25.1 dB. Within the blue boxes, the proposed method was unable to fully reconstruct the signal between the retinal layer; however, BM3D was able to maintain the data. Yet visually BM3D still shows speckle noise and noise artifacts [indicated by the white arrow in Fig. 9(c)]. Focusing on deep learning denoising methods, SiameseGAN blurred the foreground with the background but attempted to remove the noise artifacts [shown in the blue box in Fig. 9(h)]. It also eliminated speckle noise, unlike DnCNN [Fig. 9(f)]. DnCNN attempted to remove noise artifacts; however, focusing on the chosen region within the blue box shows that DnCNN sharpened the image and added speckle noise as data within the retinal layers. SE performed similar [Fig. 9(g)] to the first image presented in Fig. 8(g). The proposed method was able to visibly remove the speckle noise and separate the foreground from background through hard thresholding, which is indicated by achieving the highest CNR of 14.2 dB. As mentioned, it was restricted within the signal retrieval in the retinal layers (Table 4).

**Table 4:** Quantitative results of state-of-the-art denoisers against the proposed method in averaged PSNR, CNR, and ENL, all in dB, and SSIM for Duke images.

NET	SSIM	PSNR	CNR	ENL
TEAR - $L_{\mathrm{Loss}}$	**0.74**	24.6	**14.2**	**1380.7**
BM3D^13^	0.62	**25.0**	13.0	640.8
WIENER^8^	0.53	15.7	9.5	530.6
NLM^7^	0.51	24.1	9.8	527.6
DnCNN^17^	0.38	15.9	4.2	612.7
SiameseGAN^20^	0.57	19.6	5.8	589.5
SE^26^	0.61	23.9	7.4	894.5

Note: bold values indicate the highest value.

Next, Table 5 displays the quantitative results with evaluation metrics of the proposed method against state-of-the-art denoisers for the dentistry dataset that is averaged over the dataset. All denoisers mentioned behaved in a similar manner on the dentistry dataset as on the DUKE dataset, and this is shown both qualitatively and quantitatively. Figure 10 displays the noisy image and the outputs of each denoiser in consideration. Initially focusing on speckle repression, NLM, DnCNN, and Wiener visually were unable to remove speckle noise. This is shown through their respective CNR and ENL values. However, all three were able to preserve the edges of the foreground. DnCNN removed useful data shown by the white arrow in Fig. 10(f) but sharpened the foreground at the edge. Wiener again was unable to differentiate between the foreground and background [Fig. 10(d)]. Weiner and SiameseGAN produced the lowest CNR of 1.2 and 2.3 dB, respectively, which proved that thresholding was not performed correctly. In addition, SiameseGAN [Fig. 10(g)] blurred the foreground as well as added data (shown in the blue box). This is supported with SSIM and PSNR values of 0.62 and 15.3 dB, respectively. On the other hand, SE was able to restore a sufficient amount of the image, indicated visually and with an SSIM value of 0.80. Visually, the leading denoisers in this comparative study were TEAR (the proposed method) and BM3D. Both performed thresholding correctly while persevering the edge of the enamel layer. However, the TEAR method blurred the data within the dental layer, whereas BM3D was able to reconstruct the data efficiently [Figs. 10(b) and 10(c)]. This is indicated through BM3D obtaining the highest PSNR of 29.7 dB. On the other hand, the proposed method achieved the highest CNR and ENL with an increase of 22% and 15%, respectively, showing a substantial enhancement of the dental information and aggressive smoothing of the background. However this was achieved at the cost of a lower PSNR (representing signal retrieval).

**Table 5:** Quantitative results of state-of-the-art denoisers against the proposed method in averaged PSNR, CNR, and ENL, all in dB, and SSIM for dentistry images.

NET	SSIM	PSNR	CNR	ENL
TEAR - $L_{\text{Loss}}$	**0.90**	27.9	**6.3**	**120.8**
BM3D^13^	0.83	**29.7**	4.9	102.5
WIENER^8^	0.62	23.8	1.2	110.9
NLM^7^	0.50	22.8	4.8	42.4
DnCNN^17^	0.40	15.9	4.2	112.7
SiameseGAN^20^	0.62	15.3	2.3	122.2
SE^26^	0.80	24.1	3.1	103.7

Note: bold values indicate the highest value.

Finally, each DL model was timed for training and testing functionalities for the dentistry and DUKE datasets, which consist of 5000 images of size 512×500  pixels and 18 sets of images with a size of 500×900  pixels, respectively. This is to evaluate if they provide results within a timely manner, focusing more on the training time taken, for adaptation of new and different datasets when required in an efficient process. Therefore, results are displayed in Table 6 for the CNN models against TEAR-$L_{\text{Loss}}$ for both datasets. Regarding the dentistry and DUKE datasets, the proposed method was tested within 22.3 and 21.2 s, respectively. This produces an average time of 21.7 s taken that is at least 6% to 10% less than other DL models. This implies that TEAR-$L_{\text{Loss}}$ is a lightweight model that has numerous hyperparameters that are subject to training for different datasets.

**Table 6:** Time taken for testing for denoising on the dentistry dataset (100 images of size 500×412  pixels) and the Duke dataset (18 sets of size 500×900  pixels) of the proposed method against well-known CNNs in seconds.

Net	Time taken (ms)
Dentistry dataset	
TEAR - $L_{\text{Loss}}$	22.3
DnCNN^22^	51.1
SiameseGAN^20^	25.5
SE^32^	19.5
Duke dataset	
TEAR - $L_{\text{Loss}}$	21.2
DnCNN^22^	32.3
SiameseGAN^20^	22.5
SE^32^	19.6

### 4.3 Classification

**Fig. 11:**
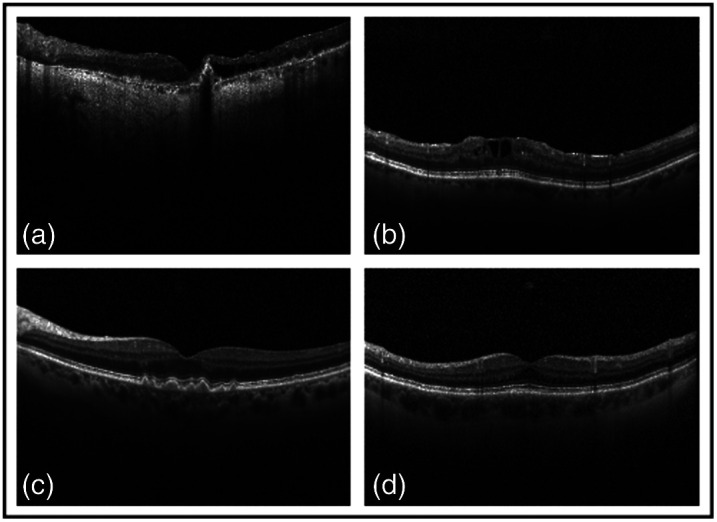
Examples of the OCT2017 dataset of each class provided: (a) CNV, (b) DME, (c) drusen, and (d) normal.

**Fig. 12:**
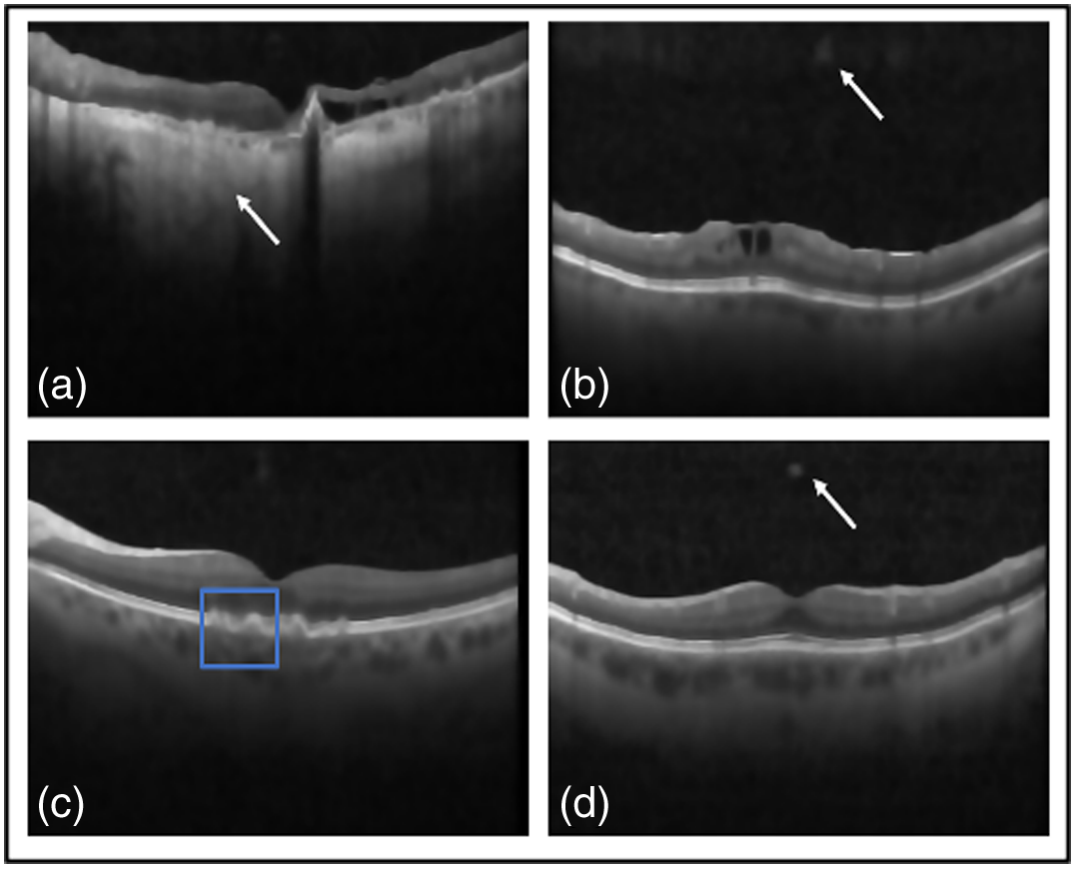
Denoised outputs of selected image from each class provided from the OCT2017 dataset, where Fig. 11 displays the corresponding noisy image. (a) CNV, (b) DME, (c) drusen, and (d) normal.

Further analysis was conducted with another public dataset called OCT2017,^35^ which consists of 84,484 B-scans of noisy images for three different diseases and normal datasets. The diseases included choroidal neovascularization (CNV), diabetic macular edema (DME), and drusen. The dataset was set with each class consisting of 37205, 11348, 8616, and 26315 images, for normal, CNV, DME and dursen, respectively. The testing dataset contains 250 images for each class. This dataset was chosen to further prove that denoising is significantly helped with classification. Figure 11 presents the different noisy images from each class. Starting with denoising the images, all images of the classes were combined and shuffled then split into training [70% (59,140 images)], validation [20% (16900 images)], and testing [10% (8448 images)]. Regarding data preparation, noisy/clean image pairs were created using the procedure for the dentistry dataset. The data augmentation mentioned in Sec. 3.1 was conducted on this dataset, and it was trained and tested with our proposed method with the optimal implementation settings acquired from the evaluation study being employed to obtain optimal results: the batch size was set to four, the optimal image size was 500  pixels×900  pixels and 500  pixels×500  pixels, the leading learning-rate was $5\times 10^{-4}$, and the number of epochs was 200. Qualitative and quantitative results of denoising for each class were averaged over the dataset, computed and displayed in Table 7 and Fig. 12.

**Table 7:** Quantitative results of denoising each class from the OCT2017 dataset using the proposed method in averaged PSNR, CNR, and ENL, all in dB, and SSIM.

NET	SSIM	PSNR	CNR	ENL
CNV	0.78	25.9	15.9	728.1
DME	0.66	21.4	6.5	333.3
Drusen	0.71	19.8	9.3	379.9
Normal	0.61	24.7	4.6	203.2
Average	0.69	22.9	9.1	411.1

By inspection, it can be concluded that our proposed method efficiently removes speckle noise and artifacts. This is indicated with the relatively high CNR of 9.1 dB, which suggests speckle repression relative to both background and signal ROIs. A leading ENL of 411.1 dB furthermore signifies smoothing of retinal layers with a minimal loss of useful data. This is pointed out by the blue box in Fig. 12(c) and the white arrow in Fig. 12(a). However, in the background of Figs. 12(b) and 8(d), the white arrows point out blurred noise artifacts that lower the SSIM and PSNR values. This is due to the limitation of not restoring the image fully because both metrics focus on both similarity and precision of the predicted image against the clean reference image.

Afterward, both noisy OCT2017 and denoised OCT2017, using the proposed method, is submitted to the augmentation process, as described in Sec. 3.1, and then into a classification CNN model. The CNN consist of three convolution blocks with activation functions of ReLU and max pooling layers in between each of them. This is followed by a dropout layer to minimize the risk of overfitting and topped with a flatten layer and a fully connected layer with ReLU activation function. Implementation settings consist of using an ADAM optimizer with a learning rate of $1\times 10^{-2}$, sparse categorical cross entropy as the loss function, 200 epochs, and a batch size of 12 for optimal results.

**Fig. 13:**
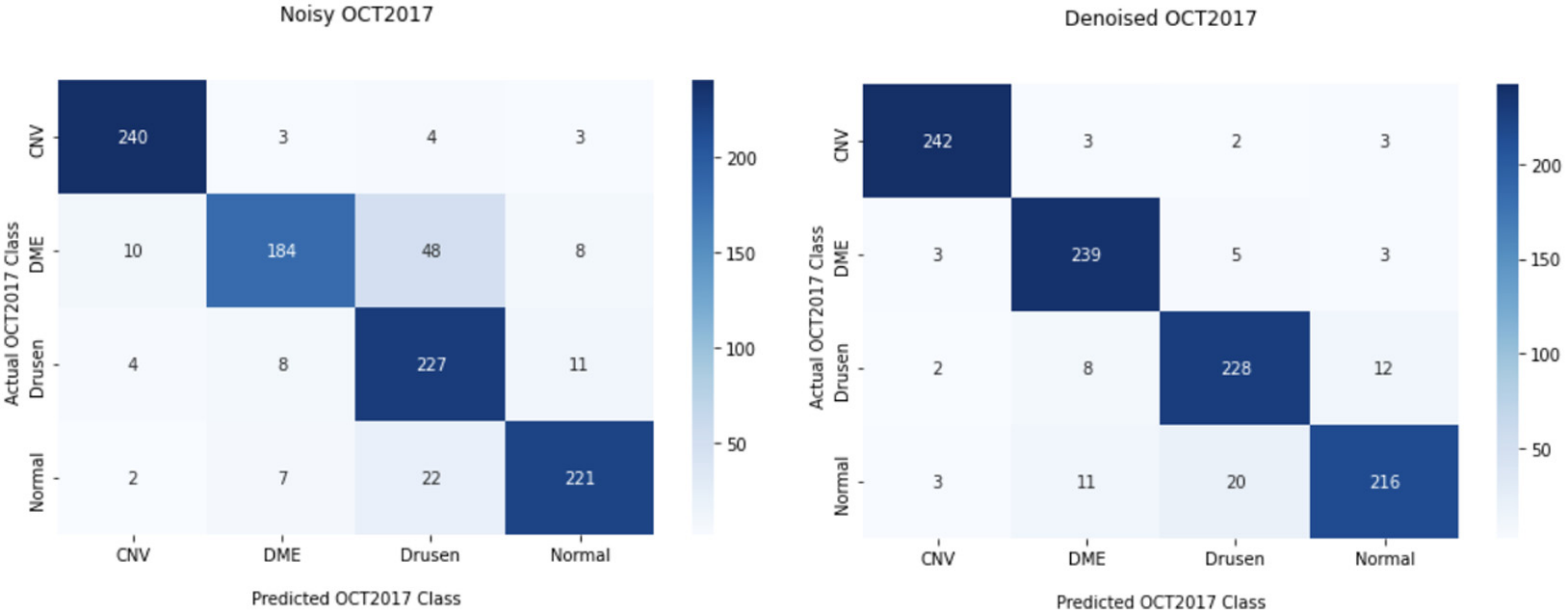
Examples of the OCT2017 dataset of each class provided: (a) CNV, (b) DME, (c) drusen, and (d) normal.

For each dataset, the classification model was evaluated using the test dataset through the accuracy and confusion matrix for quantitative evaluation. Confusion matrices are displayed in Fig. 13; these show the numerical metrics of accuracy, specificity, sensitivity, precision, and F1 score, which are mathematically expressed as

$$\text{Accuracy}=\frac{\mathrm{TN}+\mathrm{TP}}{\mathrm{TN}+\mathrm{FP}+\mathrm{TP}+\mathrm{FN}},$$ (18)

$$\text{Specificity}=\frac{\mathrm{TN}}{\mathrm{TN}+\mathrm{FP}},$$ (19)

$$\text{Sensitivity}=\frac{\mathrm{TP}}{\mathrm{TP}+\mathrm{FN}},$$ (20)

$$\text{Precision}=\frac{\mathrm{TP}}{\mathrm{FP}+\mathrm{TP}},$$ (21)

$$F1\;\text{score}=2\times \frac{\text{Precision}\times \text{sensitivity}}{\text{Precision}+\text{sensitivity}}.$$ (22)

where TP and FP are true positives and false positives, respectively. TN and FN are true negatives and false negatives, respectively. Specificity is also called true negative rate, and sensitivity is sometimes called true positive rate or recall, both of which are tests of the ability to correctly identify the correct classification. Precision is a ratio of accurate classification against all positive classifications (both true and false). The F1 score is a better measure to provide a sustainable balance between precision and sensitivity as well as to provide a metric if there is a class imbalance. However, this is not the case for the testing dataset, but an imbalance is shown in the training dataset. Outcomes of both noisy and denoised datasets are displayed in Table 8.

**Table 8:** Quantitative results of the classification task with noisy OCT2017 and denoised with TEAR OCT2017 datasets in averaged accuracy, sensitivity, specificity, precision, and F1 score over all classes.

Metric	Noisy OCT2017	Denoised OCT2017
Accuracy	87.2%	92.6%
Sensitivity	0.87	0.94
Specificity	0.96	0.97
Precision	0.88	0.91
F1 score	0.87	0.92

In comparing numerical outputs of noisy and denoised OCT2017, the denoised dataset improved all of the metrics computed. The sensitivity had an increase of approximately 8% and a specificity with an increase of 1%, as well as a 3% and 5% increase in precision and accuracy, respectively, signifying a more stable classification model that is reliable for accurately classifying augmented images. This demonstrates the value of image denoising of OCT images to do any further processing tasks, such as classification, detection, and segmentation. This is shown by the increase in all quantitative metrics of image classification after denoising the dataset using the proposed method.

## 5 Discussion

Traditional denoising program are NLM,^7^ Wiener,^8^ and the most established one, BM3D,^15^ and these are applied to the SD-OCT data. One of the main advantages is that none of them needs reference images for denoising. Nonetheless, drawbacks include losing meaningful data around retinal and dental layers through resultant heavy smoothing or limited noise removal. Additionally, processing is time consuming when a large dataset is employed.

Recently, deep learning (DL) methods have been implemented for many image processing tasks, such as classification, segmentation, and denoising. Numerous CNN layouts were implemented for low-dose CT and MRI.^36^ Nonetheless, there is limited research focused on denoising SD-OCT datasets. The leading DL models are DnCNN,^22^ hybrids of GAN (such as SiameseGAN^20^ and WGAN^21^), and autoencoder (hybrids of AE, such as SE^26^).^25^ These were implemented on public SD-OCT datasets, such as Duke^14^ and Topcon,^21^ that are retinal images. DL methods were either not compared against other popular DL methods or proven to improve the next-step analysis of images. Next-step analysis examples include retinal layer segmentation, retinal diseases image classification, and caries detection. Most importantly, no clinician input was given to the amount of data removed or added. Further, there is yet to be a DL model implemented for denoising SD-OCT dental images. Hence, the proposed method here is the first work undertaken to show efficacy through comparison against state-of-the-art classical denoising methods, deep learning models, and further analysis of the next image processing tasks (classification). A key task is denoising more than one dataset in different medical fields, both ophthalmology and dentistry.

The proposed method has several advantages that are distinctive and shown in an ablation study. This is displayed in Tables 1–3 and Figs. 4–7. First, it deploys AGs into the data augmentation operation to provide the model with foreground ROIs. This allows the model to focus on hard thresholding of the image, as well as creating a larger dataset from the limited data provided from both medical fields. The proposed framework includes data preparation to create clean reference images using BM3D to ensure the minimal amount of removal of useful data. Next, the model is a new hybrid of ViT, implemented as an encoder in an autoencoder to utilize the attention score from AGs and correlate the ROIs to reconstruct the image in the correct manner. Specifically, this did not include the addition of data due to any realignment issue or noise artifacts or the removal of useful data between the retinal or dental layers. Another addition was a new loss function that combined multiple image quality metrics, such as PSNR, MSE, and CNR, with structural difference between ROIs. Each metric respectively focused on signal restoration, error in data retrieval, thresholding, and edge preserving. This creates a robust framework because different types of noise artifacts and speckle noise were removed to an appropriate limit without removing useful data.

Two datasets were trained and tested; one was a public retinal dataset [14] that consisted of noisy/clean image pairs, and the second was a dental dataset that consisted of noisy images. It is important to mention that the proposed framework created reference images and was able to improve denoised results for the dental dataset. This is displayed in Table 5 and Fig. 10. However, creating reference images using BM3D is not an optimal solution. Quantitative and qualitative metrics for BM3D support this statement in Table 5 and 6. Our proposed method is unique in that it produces highly accurate denoised results without the need for a large B-scan volume dataset. Even with a limited amount of data, the user can achieve remarkable accuracy by utilizing a sophisticated approach that involves the meticulous averaging of B-scans. This means that our method does not impose the traditional requirement for a large-scale dataset, making it especially useful in situations for which obtaining extensive data may be difficult or resource intensive. What distinguishes our approach is its ability to extract optimal denoised images efficiently, which is accomplished without the need for a reference image. This not only streamlines the denoising process but also highlights the versatility of our method, demonstrating its ability to deliver superior results within a reasonable timeframe, making it ideal for applications in which data availability is limited.

SDOCT images in ophthalmology are typically analyzed for different types of diseases, such as DR,^2^ AMD,^3^ CNV, DME, and drusen.^35^ All of these are commonly spotted within the retinal layer. In this paper, it has been demonstrated that denoising the public dataset^35^ allows for a higher accuracy at classifying three different diseases in OCT images (Table 8). In the future, clinicians will be queried as to whether the proposed method restores the images effectively for them to diagnose and specifically if they aid in detecting carries in the dentistry dataset.

## 6 Conclusion

This paper proposed a framework that effects denoising and speckle-reduction and improves the SNR for OCT images in the medical field. The OCT images were captured by SD-OCT for the ophthalmology and dentistry fields. This delivers substantial advantages to clinicians because it maintains useful information to aid in clear and unambiguous diagnosis. The proposed method starts with overcoming the first obstacle of employing OCT by supplementing the limitation of clean OCT datasets through data augmentation. This aids in optimizing the supervised learning within the architecture. The augmented data is fed into an autoencoder that has a transformer (ViT) as an encoder and a simple CNN for decoder. The ViT provides foreground ROIs correlated with neighboring regions. This aids the autoencoder in restoring the image efficiently and maintaining the layers of both retinal and dental data for clinicians. The proposed method improved the PSNR by 27.9 dB, CNR by 6.3 dB, SSIM of 0.9, and ENL by 120.8 dB for the dental dataset and by 24.6 dB, 14.2 dB, 0.74, and 1038.7 dB, respectively, for the retinal dataset. Through testing multiple datasets, the framework as demonstrated to have the ability to be applied to different types of OCT images in dentistry and ophthalmology as it is capable of adapting automatically to different datasets, especially with OCT images in different medical fields. In future work, this denoising methodology will be conducted on dermatology and cardiology datasets. Further work will also simulate the effect of further innovations (segmentation and classification) as well as the creation of an end-to-end denoising and detection framework for clinicians utilizing OCT.

## Data Availability

All relevant code and data are available upon request.
